# Genomic characterization and outbreak investigations of methicillin-resistant *Staphylococcus aureus* in a county-level hospital in China

**DOI:** 10.3389/fmicb.2024.1387855

**Published:** 2024-04-04

**Authors:** Linyao Huang, Liangrong Zhu, Jianxin Yan, Yajing Lin, Ding Ding, Long He, Yexuzi Li, Yi Ying, Lijiong Shen, Yuhan Jiang, Haijun Cai, Tian Jiang

**Affiliations:** ^1^Department of Clinical Laboratory, The First People's Hospital of Wenling, Affiliated Wenling Hospital, Wenzhou Medical University, Wenling, China; ^2^Department of Pharmacy, Wenling Hospital of Traditional Chinese Medicine, Affiliated Wenling Traditional Chinese Medicine Hospital, Zhejiang Chinese Medical University, Wenling, China; ^3^Department of Critical Care Medicine, The First People's Hospital of Wenling, Affiliated Wenling Hospital, Wenzhou Medical University, Wenling, China; ^4^Department of Traditional Chinese Medicine, The Affiliated Xianju’s Hospital, Hangzhou Medical College, Xianju, China; ^5^School of Public Health, Hangzhou Medical College, Hangzhou, China; ^6^Burn Unit, The First People's Hospital of Wenling, Affiliated Wenling Hospital, Wenzhou Medical University, Wenling, China

**Keywords:** MRSA, whole-genome sequencing, molecular epidemiology, transmission, nosocomial outbreak, country-level hospitals

## Abstract

Methicillin-resistant *Staphylococcus aureus* (MRSA) is a common pathogen contributing to healthcare-associated infections, which can result in multiple sites infections. The epidemiological characteristics of MRSA exhibit variability among distinct regions and healthcare facilities. The aim of this study was to investigate the molecular epidemiology and nosocomial outbreak characteristics of MRSA in a county-level hospital in China. A total of 130 non-repetitive MRSA strains were collected from December 2020 to November 2021. Whole-genome sequencing (WGS) was performed to identify antimicrobial resistance and virulence factors. Phylogenetic analysis was conducted to ascertain genetic diversity and phylogenetic relationships. Independent transmission scenarios were determined by the phylogeny derived from single nucleotide polymorphisms (SNPs) within the core genome. All the MRSA isolates were collected from the intensive care unit (30.00%, 39/130), the department of otorhinolaryngology (10.00%, 13/130) and the department of burn unit (9.23%, 12/130). The clinical samples mainly included phlegm (53.85%, 70/130), purulent fluid (24.62%, 32/130), and secretions (8.46%, 11/130). The resistance rates to erythromycin, clindamycin and ciprofloxacin were 75.38, 40.00, and 39.23%, respectively. All the isolates belonged to 11 clonal complexes (CCs), with the major prevalent types were CC5, CC59, and CC398, accounting for 30.00% (39/130), 29.23% (38/130), and 16.92% (22/130), respectively. Twenty sequence types (STs) were identified, and ST59 (25.38%, 33/130) was the dominant lineage, followed by ST5 (23.84%, 31/130) and ST398 (16.92%, 22/130). Three different SCCmec types were investigated, most of isolates were type IV (33.85%, 44/130), followed by type II (27.69%, 36/130) and type III (0.77%, 1/130). The common clonal structures included CC5-ST5-t2460-SCCmec IIa, CC59-ST59-t437-SCCmec IV and CC398-ST398-t034-SCCmec (−), with rates of 16.92% (22/130), 14.62% (19/130), and 13.84% (18/130), respectively. Only 12 panton-valentine leucocidin (PVL) positive strains were identified. Two independent clonal outbreaks were detected, one consisting of 22 PVL-negative strains belongs to CC5-ST5-t2460-SCCmec IIa and the other consisting of 8 PVL-negative strains belongs to CC5-ST5-t311-SCCmec IIa. Overall, our study indicated that the CC5 lineage emerged as the predominant epidemic clone of MRSA, responsible for nosocomial outbreaks and transmission within a county-level hospital in China, highlighting the necessity to strengthen infection control measures for MRSA in such healthcare facilities.

## 1 Introduction

Methicillin-resistant *Staphylococcus aureus* (MRSA) is the predominant pathogen responsible for hospital-acquired infections. It can lead to various diseases, including wound infections, food poisoning, pneumonia, infective endocarditis, sepsis, and other systemic infections (Patel and Rawat, 2023). MRSA infections are characterized by prolonged hospital stays and a high mortality rate. In the United States, the mortality rate attributed to hospital-acquired MRSA infections is 14.2%, and the associated economic costs amount to US$3–4 billion annually (Kim et al., 2018; Nelson et al., 2022). The prevalence and epidemiology of MRSA are continuously evolving, with newly adapted MRSA clones emerging as predominant strains in various geographical regions. Therefore, the prevention and control of MRSA become a major global public health challenge.

Analyzing the genetic characteristics of MRSA is an effective approach to understand its evolution and transmission mechanisms (Giulieri et al., 2020), thereby accordingly preventing and controlling the MRSA infection (Lee et al., 2018). Currently, genetic typing methods for MRSA, including multilocus-sequence typing (MLST) (Jin et al., 2022), identification staphylococcal cassette chromosome mec (SCCmec), sequencing of the highly polymorphic repeat region of *Staphylococcus aureus* protein A (spa) gene (Tsergouli et al., 2022), and toxin genes such as panton-valentine leucocidin (PVL) (Zhu Y. et al., 2022).

The genetic characteristics and virulence gene profiles of MRSA have been associated with the antimicrobial susceptibility, transmission capability, length of hospitalization, mortality rate and prognosis (Li et al., 2019). For example, compared to other staphylococci, CC5 and CC8 lineages exhibit a higher antibiotic resistance rate (Jin et al., 2022). In recent years, ST5 strains have shown a higher level of resistance to fosfomycin, with the resistance rate increasing from 19.5 to 67.3% (Chen et al., 2022). According to pathological examination results, ST59-t437 strains primarily target the host’s lungs, leading to severe inflammatory reactions, tissue damage, and the substantial exudation of inflammatory mediators and cells (Liao et al., 2021). In addition, studies have suggested that SCCmec III strains tend to carry the highest number of resistance genes and exhibit the highest rates of multidrug resistance. SCCmec IV and V strains display increased sensitivity to ceftobiprole (BPR) (Zhu F. et al., 2022). Furthermore, compared to spa t037, spa t002 exhibits lower sensitivity to daptomycin and rifampicin (Wang et al., 2022). Moreover, PVL can lead to tissue necrosis and leukocyte lysis, and it is considered a significant contributor to skin and soft tissue infections. MRSA is considered to be a major nosocomial infection pathogen, PVL-positive CC5 strains have reported to be associated with nosocomial outbreaks, demonstrating a trend of global dissemination (Lindner et al., 2022; Aloba et al., 2023).

The genetic characteristics of MRSA is diverse in different regions, hospitals and time periods. In European countries, CC5 and CC22 are the predominant clonal complexes associated with bloodstream infections; in Latin America, CC8 is the major epidemic clone; and in Asia CC8 is the prevalent clone, while in East Asia CC5 is the primary epidemic clone (Aires-de-Sousa, 2017). A survey, conducted across 22 tertiary hospitals in China, revealed that the predominant epidemic clones was CC59 (33%, 154/471), CC239 (25%, 116/471), and CC5 (21%, 96/471), indicating a high prevalence of CC59 in China (Chen et al., 2022). Furthermore, it could be a result of clonal replacement. For instance, a study from a tertiary hospital in China found that ST239-t030-SCCmecIII rapidly replaced ST239-t037-SCCmecIII as the predominant epidemic clone in this hospital since 2000 (Chen et al., 2010). Meanwhile, the latest multi-center survey report from China indicates a significant increase in the isolation of MRSA ST59 clones from bloodstream infections (Jin et al., 2021), CC59-ST59-t437-SCCmecIV has become the predominant epidemic strain (Wang et al., 2022).

Although several studies have delved into the molecular epidemiological characteristics of MRSA strains in China, the aforementioned research predominantly centers on tertiary and teaching hospitals, leaving a gap in understanding the genetic traits of MRSA in county-level hospitals. China is one of the most populous countries in the world, in which a half of the population resided in rural areas, and sought medical care in nearly 11,000 county-level hospitals nationwide (Dong et al., 2019). Therefore, it is particularly important to conduct relevant research for developing tailored strategies of MRSA prevention and control in county-level hospitals. To address this concern, the genomic characterizations and epidemiological surveillance of 130 MRSA isolates recovered from a county-level hospital in this study were investigated.

## 2 Materials and methods

### 2.1 Sample collection

A total of 130 non-duplicate MRSA strains were isolated from Affiliated Wenling Hospital of Wenzhou Medical University from December 2020 to November 2021. These samples were obtained from various sources including blood, pus, and sputum, etc. All samples were delivered to the microbiology laboratory with optimal transport and incubated at 37°C on Columbia blood agar plates for 16–18 h. Matrix-Assisted Laser Desorption/Ionization Time-of-Flight Mass Spectrometry (MALDI-TOF-MS, ANTU, China) was used to identify target strains. For strains identified as *Staphylococcus aureus*, antimicrobial susceptibility testing was performed to identify MRSA. Following CLSI 2021 standards, strains with oxacillin minimum inhibitory concentration (MIC) values > 4 μg/mL were selected as MRSA and stored at −80°C. The protocol in this study was approved by the Ethics Committee of Affiliated Wenling Hospital of Wenzhou Medical University (KY-2021-1018-01).

### 2.2 Antibiotic susceptibility testing

Antimicrobial susceptibility testing was conducted using the DxM MicroScan WalkAway ID/AST system. The antibiotics tested included: FOX (cefoxitin), OXA (oxacillin), ERY (erythromycin), CLI (clindamycin), CIP (ciprofloxacin), TET (tetracycline), GEN (gentamicin), RIF (rifampicin), SXT (sulfamethoxazole/trimethoprim), QD (quinupristin-dalfopristin), DAP (daptomycin), LNZ (linezolid), and VAN (vancomycin). Antibiotic MIC results were tested using MicroScan Panels (Beckman Coulter, Inc., Calif., United States). The resistance criteria were based on the Clinical and Laboratory Standards Institute (CLSI) 2021 guidelines (CLSI, 2021). *Staphylococcus aureus* ATCC 29213 was used as standard bacterial strain.

### 2.3 Whole-genome sequencing

Genomic DNA was extracted using the QIAamp DNA Mini Kit (Qiagen, Hilden, Germany). Sequencing was conducted using the long-read Oxford Nanopore MinION (Nanopore, Oxford, UK) and Illumina NovaSeq 6000 platforms (Illumina, Inc., San Diego, CA, United States). The resulting reads were assembled using Unicycler v.0.4.8 software (Wu et al., 2023). The BacWGSTdb 2.0 server was employed for multi-locus sequence typing (MLST) and identification of antimicrobial resistance genes (Feng et al., 2021). For Spa and SCCmec typing analysis, spaTyper 1.0 and SCCmecFinder 1.2 were utilized, respectively. Virulence gene detection was conducted using ABRicate v1.0.0. PHYLOViZ v2.0, employing the geoBURST Full MST algorithm, was utilized to assign all isolates to different clonal complexes (CCs) based on sharing six out of the seven alleles with the founder sequence type (Jin et al., 2021).

### 2.4 Phylogenetic analysis

The phylogenetic tree generated by cgSNP analysis was performed using Snippy v4.6.0. The SNPs between each pair of isolates in different groups were calculated by snp-dists v0.8.2, and the recombination events were calculated by Gubbins v3.3.2 based on the alignments. Fasttree v2.1.11 infers an approximately-maximum-likelihood phylogenetic tree from these non-recombinant SNPs. The visualization and annotation of phylogenetic trees, along with the identification of antimicrobial resistance genes, sequence type, clonal complex, Spa type, Sccmec type, PVL type and strain metadata, were carried out using the Interactive Tree of Life (iTOL) V6 web server (Wu et al., 2023).

### 2.5 Statistical analysis

Statistical analysis was performed using GraphPad Prism 9.5 (GraphPad Software, United States). Timelines of the infectious patients were created by Flowchart Maker & Online Diagram Software (Draw.io, Switzerland).

## 3 Results

### 3.1 Clinical characteristics of MRSA strains

**Figure 1:**
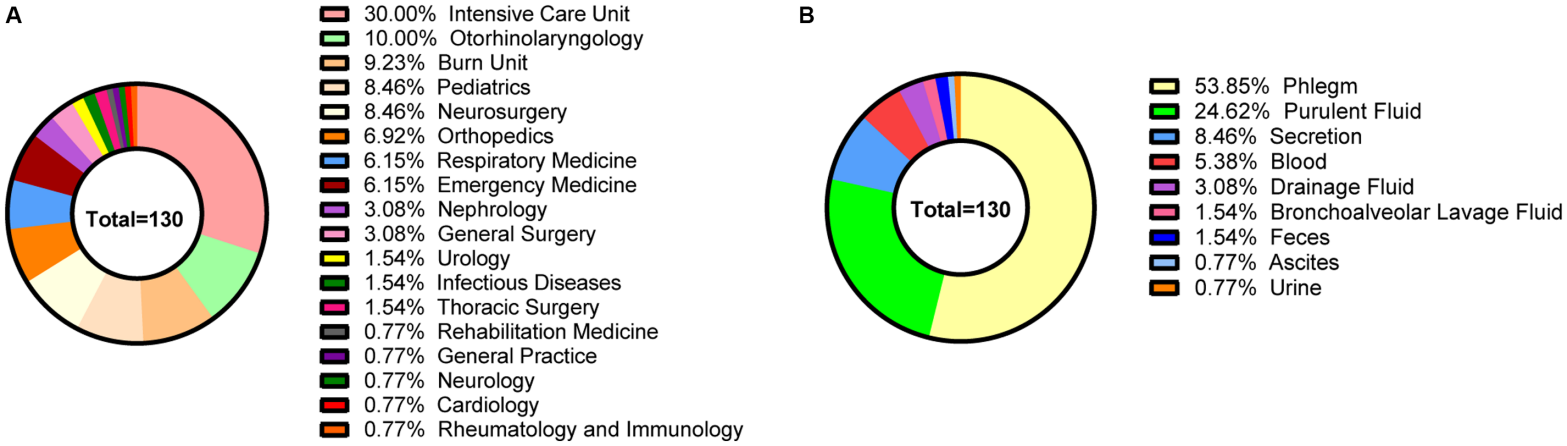
The clinical characteristics of 130 MRSA strains in this study. **(A)** The departmental distribution of all strains. **(B)** The specimen sources of all strains. Different colors represent different departments or sample types.

From December 2020 to November 2021, a total of 130 non-repetitive MRSA strains were collected. The average age of the inpatients was 51.53 ± 27.23 years. Among the patients, 57.69% (75/130) were male, while 42.31% (55/130) were female. The distribution among departments included the intensive care unit (30.00%, 39/130), the department of otorhinolaryngology (10.00%, 13/130), the burn unit (9.23%, 12/130), the pediatrics department (8.46%, 11/130), the neurosurgery department (8.46%, 11/130), and other departments (14.62%, 19/130) (Figure 1A). The specimen sources were phlegm (53.85%, 70/130), purulent fluid (24.62%, 32/130), secretions (8.46%, 11/130), blood (5.38%, 7/130), drainage fluid (3.08%, 4/130), and others (4.62%, 6/130) (Figure 1B). The patients were diagnosed as pulmonary infection (59.23%, 77/130), skin infection (16.92%, 22/130), otitis externa (6.15%, 8/130), otitis media (3.85%, 5/130), soft tissue infection (3.08%, 4/130), neonatal sepsis (1.54%, 2/130), diabetic foot ulcers (1.54%, 2/130), mastitis (1.54%, 2/130), catheter-related bloodstream infection (1.54%, 2/130), urinary tract infection (1.54%, 2/130), peritonitis (0.77%, 1/130), gastrointestinal infection (0.77%, 1/130), tonsillitis (0.77%, 1/130) and sinusitis (0.77%, 1/130).

### 3.2 Antimicrobial susceptibility testing data

**Figure 2:**
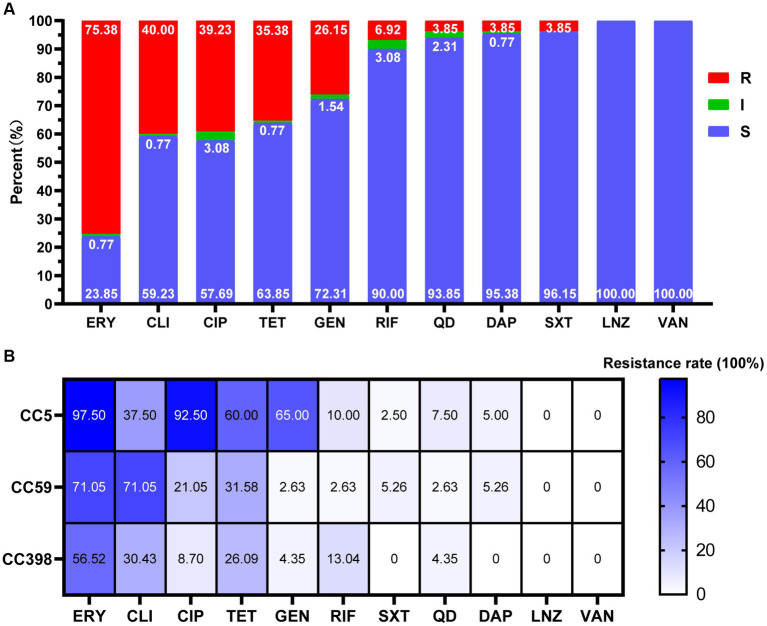
Antimicrobial susceptibility of MRSA strains in this study. **(A)** Antimicrobial susceptibility of 130 MRSA strains. R, resistance; I, intermediate; S, susceptible. **(B)** Antimicrobial resistance profiles among different CCs (CC5, CC59, CC398). The cell in different colors represents the rates of drug resistance. As the color deepens, the drug resistance rate increases correspondingly. ERY, erythromycin; CLI, clindamycin; CIP, ciprofloxacin; TET, tetracycline; GEN, gentamicin; RIF, rifampicin; SXT, sulfamethoxazole/trimetho; QD, quinupristin-dalfopristin; DAP, daptomycin; LNZ, linezolid; VAN, vancomycin.

All MRSA isolates were resistant to cefoxitin. The resistance rates to erythromycin, clindamycin, ciprofloxacin, tetracycline, gentamicin, were 75.38, 40.00, 39.23, 35.38, and 26.15%, respectively. The resistance rates for rifampicin, quinupristin-dalfopristin, sulfamethoxazole/trimethoprim, and daptomycin were relatively low, ranging from 3.85 to 6.92%. None of the isolates were resistant to linezolid and vancomycin (Figure 2A). Overall, the highest resistance rate was observed in CC5, and the next were CC59 and CC398. Their resistance rates to erythromycin were 97.50, 71.05, and 56.52%, to ciprofloxacin were 92.50, 21.05, and 8.70%, to gentamicin were 65.00, 2.63, and 4.35%, to clindamycin was 37.50, 71.05, and 30.43%, to tetracycline was 60.00, 31.58, and 26.09%, respectively. CC5 showed highest resistance rates to all antimicrobial agents, except for clindamycin, to which CC59 showed the highest resistance rate (Figure 2B).

### 3.3 Phylogenetic analysis

**Figure 3:**
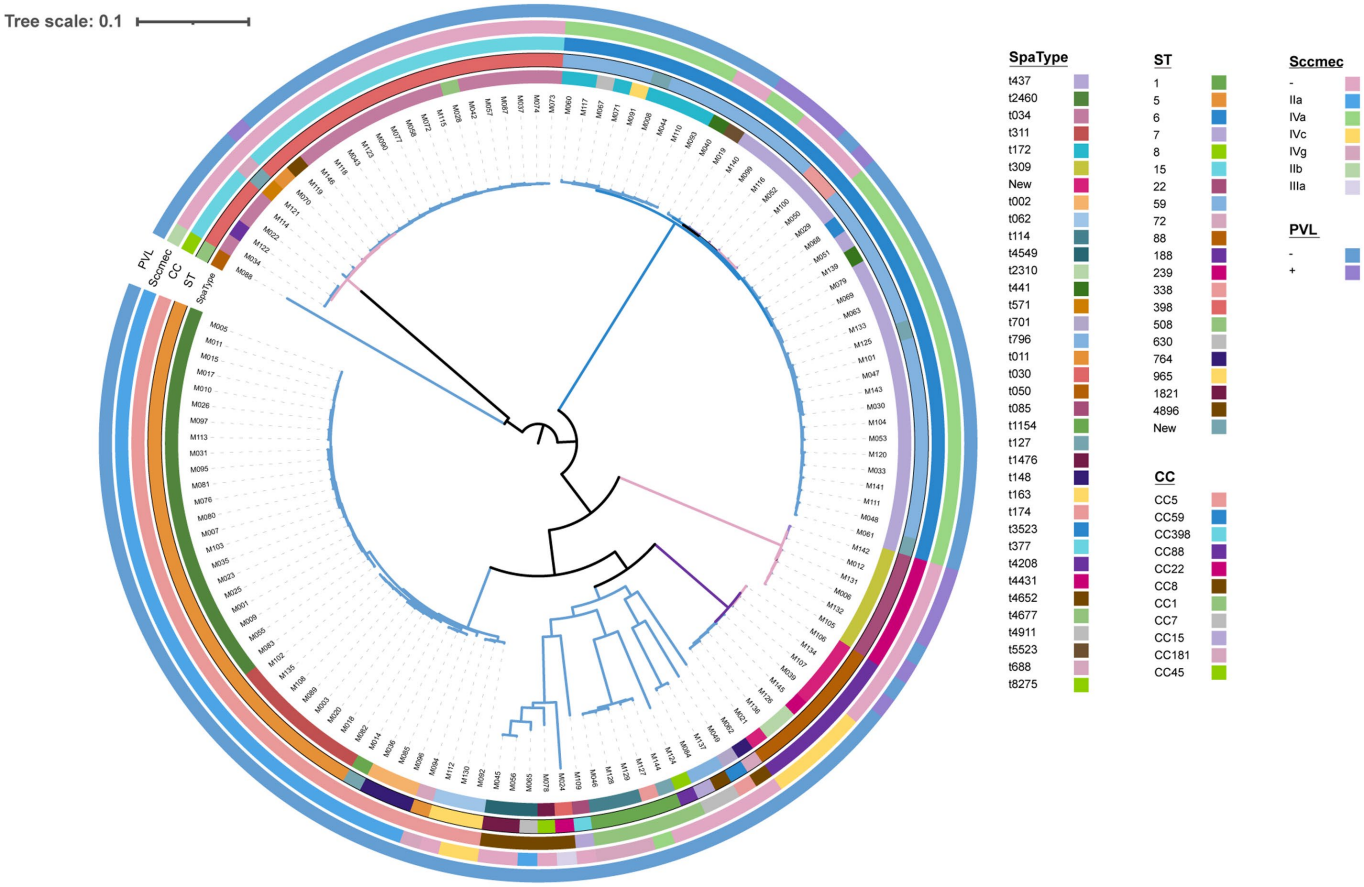
Phylogenetic tree of all 130 MRSA. Spa types, ST types, clonal complex types, SCCmec type, and PVL presence are color-coded in the inner rings.

Thirty-five spa types were identified, in which t437, t2460, and t034 were the most prevalent gene types, with the rates of 19.23% (25/130), 16.92% (22/130), and 13.85% (18/130), respectively. Twenty sequence types (STs) were observed, with the dominant lineages: ST59 at 25.38% (33/130), ST5 at 23.84% (31/130), and ST398 at 16.92% (22/130). The isolates were categorized into 11 clonal complexes (CCs). Among them, CC5 (30.00%, 39/130), CC59 (29.23%, 38/130), and CC398 (16.92%, 22/130) were the major prevalent types. In terms of SCCmec types, three distinct variations were examined--type IV 33.85% (44/130), type II 27.69% (36/130), and type III 0.77% (1/130). In this study, SCCmec type IV includes subtype IVa 25.38% (33/130), IVc 5.38% (7/130), and IVg 3.08% (4/130), SCCmec type II is composed of two main subtypes, IIa 26.92% (35/130) and IIb 0.77% (1/130). Non-typeable SCCmec types occurred in 49 isolates. Only 9.23% (12/130) Panton-Valentine leucocidin (PVL) positive strains were identified. The common clonal structures included CC5-ST5-t2460-SCCmec IIa, CC59-ST59-t437-SCCmec IV, and CC398-ST398-t034-SCCmec(−), with rates of 16.92% (22/130), 14.62% (19/130), and 13.84% (18/130), respectively (Figure 3).

### 3.4 Nosocomial outbreaks

Taking advantage of whole-genome sequencing, two clusters of nosocomial outbreaks were identified. One consisting of 22 PVL-negative strains belongs to CC5-ST5-t2460-IIa, another consisting of 8 PVL-negative strains belongs to CC5-ST5-t311-IIa.

**Figure 4:**
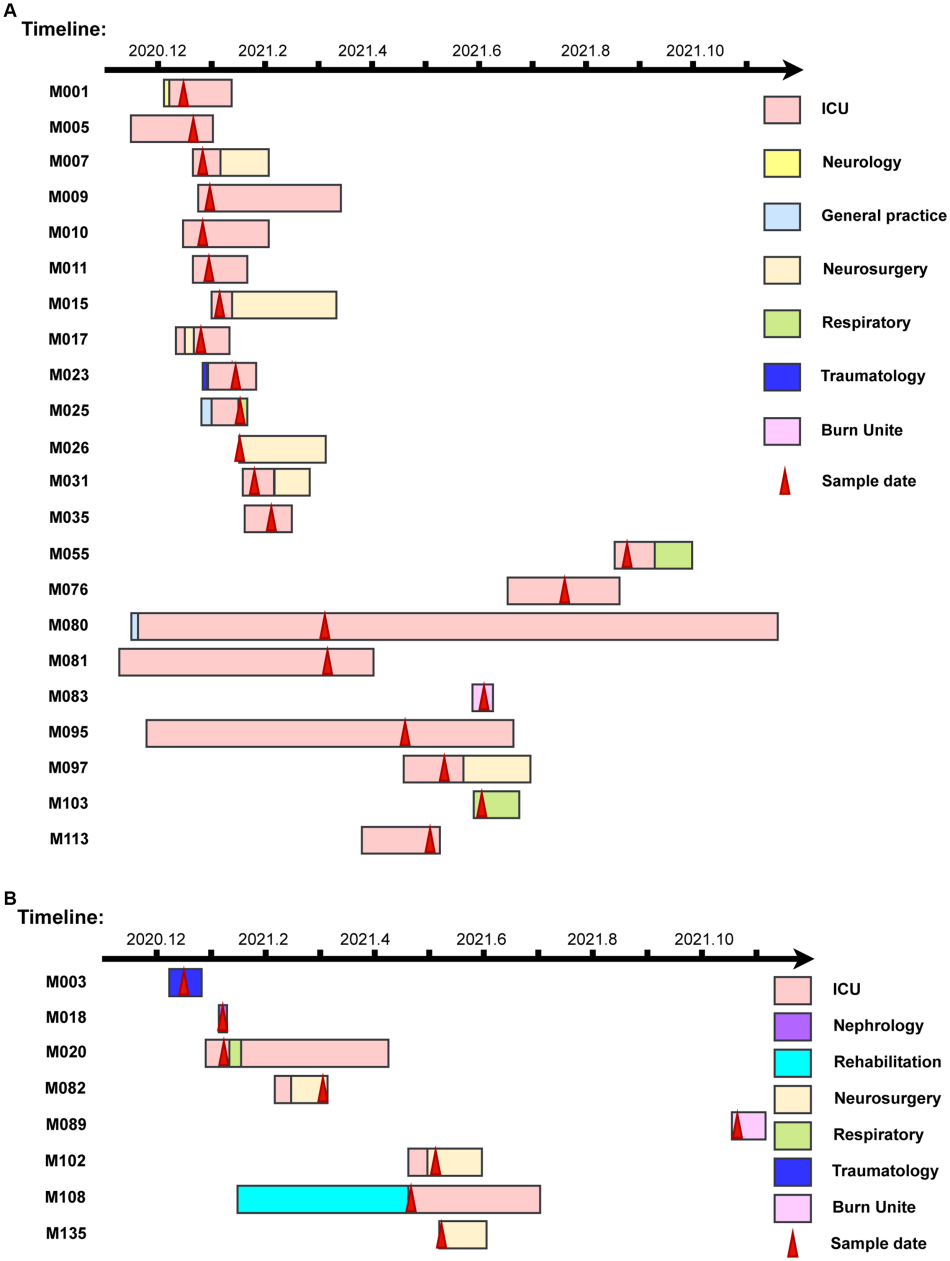
The timelines of nosocomial infection outbreaks in a county-level hospital. **(A)** The timelines of the infectious patients in Clade 1. **(B)** The timelines of the infectious patients in Clade 2. The rectangle on the timeline represents the duration of patients’ admissions. Different wards are depicted in various colors, while red arrows indicate sampling dates.

In the first outbreak cluster, 22 patients were identified. Except for M026, M083 and M103, all of the rest 19 patients had a history of ICU admission. Specifically, M080 and M095 had ICU stays for more than 6 months, with infection histories more than 2 months since the first strain infections. Samples were collected from M005, M007, M009, M010, and M011 within 1 week. In the first outbreak cluster, the period spanned 215 days from the sampling day of the first strain to the sampling day of last strain (Figure 4A). In the second outbreak cluster, there were a total of 8 patients. The samples of M020, and M108 were taken in the ICU. The samples of M082, M102, and M135 were taken from the neurosurgery department. M020 and M082 had overlapping ICU stays, suggesting a transmission of drug-resistant bacteria from M020 to M082, and subsequently to M102 and M135 (Figure 4B).

**Figure 5:**
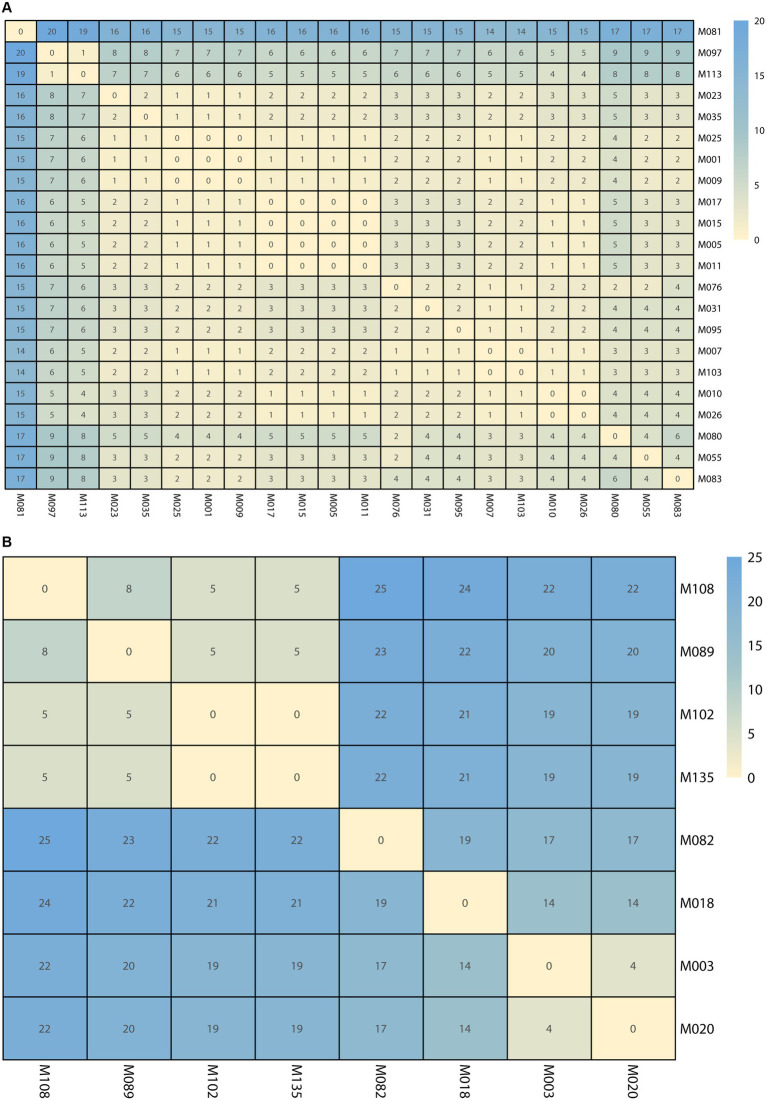
The single nucleotide polymorphisms (SNPs) numbers between each isolate. **(A)** The SNPs numbers in Clade 1. **(B)** The SNPs numbers in Clade 2. The cell in different colors represents the number of SNPs.

SNP analysis revealed nucleotide sequence differences ranging from 0 to 20 in the first cluster and 0 to 25 in the second cluster. Five pairings (M010 and M026; M005, M011, M015, and M017; M007 and M103; M102 and M135; M001, M009, and M025) had no SNP differences, suggesting that these pairs of isolates were strictly clonal (Figure 5).

In Clade 1 and Clade 2, all strains exhibited 100.00% resistance rates to FOX, OXA, ERY, and CIP. Strains from Clade 1 were all susceptible to RIF, QD, DAP, LNZ, and VAN, while Strains from Clade 2 were all susceptible to SXT, LNZ, and VAN. Compared to Clade 2, Clade 1 exhibited higher resistance rates to TET (90.91% vs. 12.50%), GEN (77.27% vs. 37.50%), and SXT (4.55% vs. 0%). Conversely, Clade 1 demonstrated lower resistance rates than Clade 2 to CLI (22.73% vs. 25.00%), RIF (0 vs. 25.00%), QD (0 vs. 25.00%), and DAP (0 vs. 12.50%). The sampling time for the first case in two outbreak clusters exceeded 12 days and 9 days from the time of admission, indicating hospital-acquired infections. The first clade had 14 more patients than Clade 2. The duration time was 57 days shorter in Clade 1. Isolates in Clade 1 were constituted strains persistently colonized in the ICU, but no further detection was observed in the last 3 months of monitoring, indicating complete clearance. On the other hand, isolates in Clade 2 spread among different departments within the hospital and were not detected again in the last month of monitoring (Figure 4).

## 4 Discussion

MRSA infection remains a global health concern, proliferating and posing significant challenges (Aloba et al., 2023). While current investigations primarily targeted tertiary and teaching hospitals (Chen et al., 2022), scant data exist regarding the genetic traits and nosocomial outbreaks of MRSA in county-level hospitals. To grasp the present landscape of MRSA prevalence, nosocomial infection outbreaks, and the molecular mechanisms involved within county-level hospitals, this study delves into the molecular characteristics of MRSA within a county-level hospital in China.

In this study, MRSA CC5, CC59, and CC398 were identified as the main prevalent lineages, with proportions of 30.00% (39/130), 29.23% (38/130), and 16.92% (22/130), respectively. Consistent with the previous studies, CC5 and CC59 are the two most predominant clones (Chen et al., 2022; Zhao et al., 2022). A recent study focused on seven representative tertiary hospitals in seven provinces and municipalities in China, including Shanghai, Zhejiang, Guangdong, Sichuan, Hubei, Jiangxi, and Inner Mongolia, from 2014 to 2020, and found that CC5 and CC59 accounted for 23.4 and 31.2%, respectively (Wang et al., 2022). An earlier study reported that CC5 and CC8 were the most common global epidemic clones, and identified CC5 as the major clone associated with hospital infections, while CC8 as the primary clone associated with community infections (Lakhundi and Zhang, 2018). Compared with CC8 MRSA, CC5 MRSA exhibited greater resistance to selective pressures, adapting and spreading in hospital environments and readily acquiring antibiotic resistance traits (Bispo et al., 2020). After 2015, CC59 gradually increased over the world, more than in China but also in countries such as the United States, Singapore, and Latin America. Most CC59 isolates showed relatively lower resistance to common antibiotics and had fewer resistance-associated fitness costs, thus demonstrating stronger competitiveness. This trend may contribute to the replacement of CC8-ST239 to CC59-ST59 (Wang et al., 2022).

Meanwhile, we identified CC398 as one of the top three clones in primary healthcare facilities in China, with a prevalence of 17.16%. Recent reports have identified CC398 as the predominant LA-MRSA clone found in livestock worldwide, including Europe, North America, Asia, and Africa (Wang et al., 2023). LA-MRSA-CC398, lacking specific host specificity, can cross various species barriers, colonize or infect animals and humans through close contact with contaminated livestock or food, leading to human infections (Tang et al., 2021; Kruger-Haker et al., 2023). Due to the stable presence of Tn916 and SCCmec in CC398, these mobile genetic elements (or other antibiotic resistance genes) are often retained when CC398 was transmitted to humans (Matuszewska et al., 2022). We speculated that the relatively high prevalence of CC398 may be associated with the well-established livestock and aquaculture industry in Wenling City, a county-level city located on the southeastern coast of Zhejiang, China.

In addition, this study found that ST59, as the most prevalent clone, accounted for 25.38% (33/130) infections, followed by ST5 and ST398, which accounted for 23.84% (31/130) and 16.92% (22/130), respectively. This indicated that in county-level hospitals in China, ST59 has become the predominant epidemic clone of MRSA at the grassroots level. These findings are in line with reports from national multicenter studies and higher-level teaching hospitals (Chen et al., 2019; Wang et al., 2022). Reports suggest that since 2010, MRSA ST59 has been on the rise in China and gradually replaced ST239 as the dominant clone in most Chinese hospitals (Jin et al., 2021). Previous studies have indicated that ST59 possesses high pathogenicity and virulence, which was considered as important reasons for its widespread dissemination of ST59 (Pimentel de Araujo et al., 2021).

Previous studies suggest that different MRSA clonal lineages were associated with their biofilm formation capabilities, the ability of the ST59 -t437-SCCmec IV clone to form robust biofilms may be the reason for its dominance and multidrug resistance in China (Zhao et al., 2022). In our study, ST59-t437-SCCmec IV accounted for 14.62% (19/130) of all MRSA, and it represented 57.58% (19/33) of all ST59 MRSA strains. Our results aligned closely with the results of a multicenter study in China (Wang et al., 2022). This indicates that ST59-t437-IV has become an important epidemic clone in China, not only in tertiary hospitals but also in county-level hospitals. In this study, ST5-t2460-SCCmec II accounts for 16.92% (22/130). Researchers have reported that in Shanghai, China, from 2008 to 2017, the prevalence of ST5-t2460 clones increased from 0 to 17.3% (Dai et al., 2019), and ST5-t2460-SCCmec II gradually became the predominant clone in multiple regions of China (Zhao et al., 2022). However, further research is required to understand how the ST5-t2460 clone gained a competitive advantage in the hospital environment. ST398-t034 accounts for 13.85% (18/130) in this study. ST398-t034 was considered to be associated with livestock transport, but has also become a major type in hospital personnel (Xu et al., 2020). This suggests the need for further monitoring the prevalence of ST398-t034 in primary healthcare facilities.

In this study, only 1 strain of ST239 was found, accounting for 0.77% (1/130). ST239 clones have traditionally been considered the major infecting strains in subacute hospital infections, persisting in the hospital environment and undergoing adaptive evolution for several decades. However, their prevalence in China sharply declined from 2008 to 2017, being replaced by the ST5-t2460 clone (Dai et al., 2019). It is noted that ST239 is no longer the prevalent clone at the county-level hospital according to our study.

The prevalence of PVL carriage varies in different regions or among different bacterial clones (Li et al., 2013). In this study, 12 PVL-positive strains were identified 9.23% (12/130), primarily consisting of C22-ST22-t309 and CC59-ST59-t437, with ratios of 41.67% (5/12) and 33.33% (4/12), respectively. A previous study in China indicated that from 2009 to 2012, the prevalence of PVL-positive MRSA strains was 28.6% (Hu et al., 2015). This difference may attribute to the fact that the patients selected for the above study were all soft tissue infections. Four hospitals in eastern China reported a PVL-positive rate of MRSA at 10.1% (Kong et al., 2018), which aligns with our results. A recent study showed that the detection rate of PVL in MRSA associated with skin and soft tissue infections (SSTIs) in China has increased from 9.09% in 2009–2011 to 22.55% in 2019–2021 (Su et al., 2023). It is imperative to intensify the surveillance of PVL-positive strains within county-level hospitals.

The antimicrobial susceptibility profile of MRSA varies in different countries and regions. In the United Kingdom, MRSA exhibited higher resistance proportions to fluoroquinolones, erythromycin, gentamicin, moxifloxacin, tobramycin and methicillin. However, the resistance rate to tetracycline was relatively low (Baede et al., 2023). The finding of this study confirmed that MRSA in Chinese county-level hospitals had a higher resistance rate to ERY, but were more sensitive to LNZ and VAN. Our results were consistent with earlier research results, MRSA in China has a higher resistance rate to oxacillin, erythromycin, and clindamycin, but lower resistance rates to linezolid and vancomycin (Fu et al., 2021; Hu et al., 2022; Wang et al., 2022). Further analysis revealed that the drug resistance profile of MRSA was associated with different clonal complexes (CCs). In our study, CC5 MRSA exhibited a resistance rate of over 90% to ERY and CIP, and over 60% to TET and GEN. CC59 MRSA was mainly resistant to OXA, ERY, and CLI, with resistance rates exceeding 70%. CC398 MRSA is primarily resistant to OXA. The results were consistent with previous studies, CC5 MRSA has the highest drug resistance rate, followed by CC59 and CC398 (Gergova et al., 2022; Zhao et al., 2022). During CC5 MRSA’s evolutionary process, it can carry more antibiotic resistance genes due to mobile genetic elements and the selective pressure of antibiotics (Challagundla et al., 2018), thereby boosting its predominance. Whole-genome sequencing not only can provide information about the genetic basis of phenotypic characteristics, including antimicrobial resistance and virulence traits, but also able to determine the single-nucleotide differences. This allows for the accurate prediction of transmission events and outbreaks (Price et al., 2013). In our study, two significant hospital-acquired outbreak events were identified, one consisting of 22 PVL-negative CC5-ST5-t2460-SCCmec IIa strains and the other comprising 8 PVL-negative CC5-ST5-t311-SCCmec IIa strains. Among the 22 strains in the first cluster, 20 patients had a history of ICU contact, except for M026 and M103. In the second cluster, among the 8 strains, except for 4 patients (M003, M018, M089, and M135), the other 4 patients had a history of ICU contact. ICUs are among the departments most vulnerable to MRSA infections and colonization. MRSA-contaminated patients and the hospital environment, particularly high-touch surfaces in patient rooms, serve as potential reservoirs for MRSA transmission (Ziegler et al., 2022). ICUs are major sources for the creation, transmission, and spread of multidrug-resistant bacteria. On one hand, increased antibiotic use when patients develop infections in the ICU leads to greater selective pressure for pathogens to develop resistance. On the other hand, ICU patients have compromised immune function, undergo mechanical ventilation, central venous catheterization and urinary catheterization procedures, and are applied invasive devices, all of which significantly increase the risk of infections in ICU patients (Pachori et al., 2019). Furthermore, factors such as inadequate equipment disinfection and sterilization, partial aerosol transmission of pathogenic bacteria, insufficient disinfection of the environment, neglect of hand hygiene, and drug-related issues further promote the spread of MRSA in hospitals (Yang et al., 2023). These results highlight the need for enhanced infection prevention and control measures in county-level hospitals in China.

This study has some limitations that needs to be acknowledged. Firstly, it is a single-center study and lacks thorough comparisons for different years. Secondly, this study lacks of correlation analysis of antibiotic resistance, virulence genes, and clinical phenotype, due to the lacks of detailed clinical information. Third, due to the lack of animal experiments, the virulence levels of some PVL-positive strains still await validation. Future research should involve multicenter longitudinal studies in county-level hospitals in China.

## 5 Conclusion

In summary, our study aimed to investigate the molecular epidemiology and nosocomial outbreak characteristics of MRSA in county-level hospitals in China. The isolates exhibited high rates of resistance to erythromycin, clindamycin, and ciprofloxacin. Molecular typing unveiled a diverse population of MRSA strains, predominantly associated with clonal complexes CC5, CC59, and CC398, among which ST59 emerged as the most prevalent sequence type. Various SCCmec types were identified, with type IV being the most prevalent. Additionally, this study detected two independent clonal outbreaks within the county-level hospital. These outbreaks were primarily composed of CC5 clone.

In conclusion, this study sheds light on the genomic features and transmission patterns of MRSA CC5 and CC59 epidemic strains within a Chinese county-level hospital, highlighting a nosocomial outbreak associated with the CC5 lineage. These findings underscore the imperative for enhanced detection and control measures for MRSA infections within county-level hospitals in China. Otherwise, the study of MRSA clonal distributions is related not only to control measures in hospitals but with also to right faster choice of therapy in cases of severe infections.

## Data availability statement

The datasets presented in this study can be found in online repositories. The names of the repository/repositories and accession number(s) can be found at: https://www.ncbi.nlm.nih.gov/, PRJNA1077681.

## Ethics statement

The studies involving humans were approved by Ethics Committee of Affiliated Wenling Hospital of Wenzhou Medical University (KY-2021-1018-01). The studies were conducted in accordance with the local legislation and institutional requirements. Written informed consent for participation was not required from the participants or the participants’ legal guardians/next of kin in accordance with the national legislation and institutional requirements.

## Author contributions

LHu: Formal analysis, Investigation, Methodology, Writing – original draft. LZ: Formal analysis, Investigation, Methodology, Visualization, Writing – review & editing. JY: Conceptualization, Data curation, Methodology, Writing – review & editing. YaL: Methodology, Project administration, Writing – review & editing. DD: Formal analysis, Methodology, Project administration, Writing – review & editing. LHe: Visualization, Writing – review & editing. YeL: Data curation, Project administration, Validation, Writing – review & editing. YY: Formal analysis, Validation, Writing – review & editing. LS: Investigation, Methodology, Writing – review & editing. YJ: Methodology, Project administration, Writing – review & editing. HC: Funding acquisition, Investigation, Methodology, Project administration, Resources, Supervision, Visualization, Writing – review & editing. TJ: Funding acquisition, Investigation, Methodology, Project administration, Resources, Supervision, Validation, Writing – original draft, Writing – review & editing.
